# Association of visceral and subcutaneous fat with bone mineral density in US adults: a cross-sectional study

**DOI:** 10.1038/s41598-023-37892-6

**Published:** 2023-07-01

**Authors:** Yanze Lin, Xugang Zhong, Dongning Lu, Wenchao Yao, Jinlei Zhou, Ruiji Wu, Fabo Feng

**Affiliations:** 1 Second Clinical Medical College, Zhejiang Chinese Medical University, Hangzhou, Zhejiang China; 2 Department of Orthopedics, Zhejiang Provincial People’s Hospital, Qingdao University, Qingdao, China; 3 Department of Orthopaedics, The First People’s Hospital of Chun’an County, Hangzhou, Zhejiang China; 4 Center for Plastic and Reconstructive Surgery, Department of Orthopedics, Zhejiang Provincial People’s Hospital (Affiliated People’s Hospital, Hangzhou Medical College), Hangzhou, Zhejiang China

**Keywords:** Public health, Endocrine system and metabolic diseases, Endocrine system and metabolic diseases

## Abstract

The relationship between the accumulation of fat in visceral or subcutaneous tissue and bone mineral density (BMD) remains unclear. Our primary objective in this study was to illuminate this relationship by conducting an investigation on a vast scale, encompassing a nationally representative population in the United States. A weighted multiple linear regression model was established to evaluate the relationship between visceral fat, subcutaneous fat, and BMD. Additionally, the exploration of the potential nonlinear relationship was conducted employing the methodology of smooth curve fitting. In order to determine potential inflection points, a two-stage linear regression model was utilized. A total of 10,455 participants between the ages of 20 and 59 were included in this study. Various weighted multiple linear regression models revealed a negative correlation between lumbar BMD and visceral mass index (VMI) and subcutaneous mass index (SMI). However, the association between VMI and lumbar BMD displayed a U-shaped pattern upon employing the smooth curve fitting, and the inflection point of 0.304 kg/m^2^was determined using a two-stage linear regression model. Our findings indicated a negative association between subcutaneous fat and BMD. A U-shaped relationship was observed between visceral fat and BMD.

## Introduction

Osteoporosis, a metabolic bone disorder characterized by the progressive reduction of bone mass, manifests as the deterioration of the intricate microstructure of osseous tissue, resulting in diminished bone strength, thereby increasing the susceptibility to low-energy or brittle fractures^1^. A staggering multitude of over 53.4 million elderly individuals in the United States suffer from the afflictions of osteoporosis and osteopenia, and it is anticipated that the incidence of these conditions will steadily surge as the population ages^2^. The fiscal burden of healthcare expenses associated with osteoporosis is projected to undergo a striking escalation of 100–200% by the year 2040, encompassing more than 2.6 million visits to medical professionals and over 500,000 hospitalizations annually^3^. The World Health Organization defines osteoporosis as a state wherein the bone mineral density (BMD) stands at least 2.5 standard deviations below the average for healthy young adults^4^. BMD serves as an approximate measure of the quantity of mineralized osseous tissue within the skeletal framework, and its decline serves as a significant precipitant of osteoporosis onset^5^. Identifying the risk factors that contribute to the reduction of BMD is of utmost importance in the preservation of bone health and the prevention of osteoporosis.

Obesity, a serious public health concern of escalating global prevalence^6^, was formerly believed to confer protection against osteoporosis; However, emerging evidence now indicates that obesity, specifically the type of adipose tissue present, may diminish BMD and heighten the risk of fractures^7^. Remarkably, the distribution of adipose tissue in localized regions, specifically subcutaneous and visceral adipose tissue, has emerged as a superior indicator of disease susceptibility when compared to overall adiposity^8,9^. While subcutaneous adipose tissue (SAT) and visceral adipose tissue (VAT) share a common gene pool^10^, their distinct structures and functions diverge^11^, instigating disparate physiological consequences within the body. Investigations conducted by the Framingham Heart study revealed that both SAT and VAT exhibited associations with metabolic risk factors, with VAT demonstrating a more robust correlation with adverse metabolic status than SAT^12,13^. Nonetheless, the relationship between VAT, SAT, and bone health remains elusive. In a prospective community-based cohort study conducted in Korea, it was found that a relatively greater volume of visceral fat and a diminished extent of subcutaneous fat might deleteriously affect bone microarchitecture^14^. A cross-sectional study suggested that excess visceral fat and subcutaneous fat could potentially exert a detrimental influence on bone health in both premenopausal and postmenopausal women^15^. However, in a study involving Chinese adults, neither visceral fat nor subcutaneous fat displayed any discernible association with BMD^16^.

Our primary aim, centered on the development of effective interventions for the prevention and management of osteoporosis, was to shed light on the relationship between VAT, SAT and bone health.

## Materials and methods

### Datasets sources

The National Health and Nutrition Examination Survey (NHANES) is a meticulously designed and methodologically rigorous multi-stage investigation that aims to evaluate health and nutritional metrics on a national scale^17^. Ethical approval was duly granted by the research ethics review board of the National Center for Health Statistics (https://www.cdc.gov/nchs/nhanes/irba98.htm). All individuals involved in the study willingly and knowingly affixed their signatures to a written informed consent form. This study was conducted in accordance with the Declaration of Helsinki (Fortaleza 2013 revision), and all methods were executed in accordance with the relevant guidelines and regulations.

To conduct our study, we utilized the NHANES 2011–2018 dataset, which includes a wealth of information about both visceral and subcutaneous fat, along with lumbar BMD. By harnessing this comprehensive dataset, we were able to conduct a robust analysis of the association between SAT, VAT and bone health.

### Participants eligible

**Figure 1:**
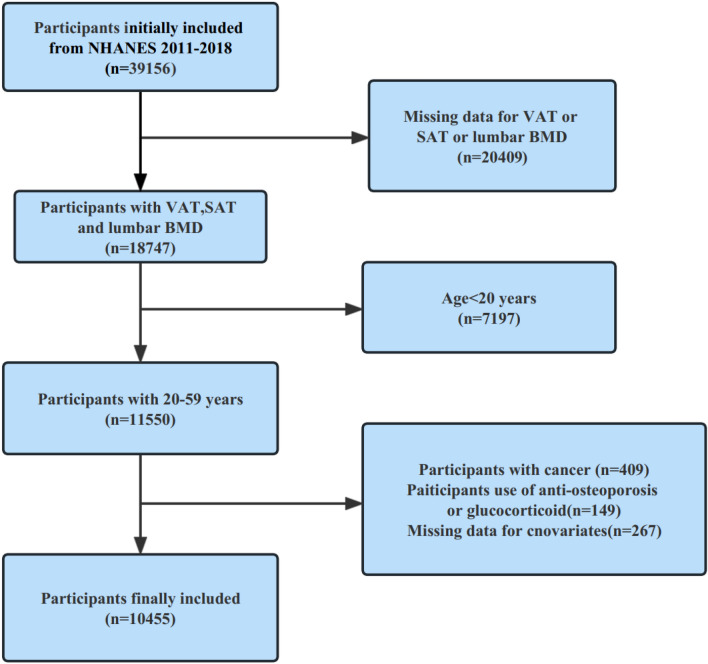
Flowchart of study participants.

Individuals falling within the following categories were excluded from participation: (1) pregnant; (2) undergone contrast media examinations within the preceding week; (3) exceeded 450 pounds in weight or surpassed a height of 6 feet 5 inches; and (4) harbored any form of implants in the body. The original cohort consisted of 39,156 participants, from which 20,409 individuals lacked essential data regarding visceral or subcutaneous fat, as well as lumbar BMD. Furthermore, 7197 participants below the age of 20, along with 409 individuals diagnosed with cancer, and 149 individuals undergoing treatment with anti-osteoporosis medication or glucocorticoids, were excluded from our analysis. In addition, certain individuals who count for a mere 5.1% representation within the entire population under study had to be excluded due to incomplete data about their height, weight, as well as serum levels of calcium, phosphorus, and vitamin D. Ultimately, a total of 10,455 participants constituted our final cohort (Fig. 1).

### DXA measurements

Dual-energy X-ray Absorptiometry (DXA) scan was meticulously performed utilizing the Apex 3.2 software on the Hologic Discovery model A densitometer (Hologic, Inc., Bedford, Massachusetts). Subsequent analysis of the scans entailed the utilization of the Hologic APEX 4.0 software, coupled with the NHANES BCA option expertly executed by radiographers who possessed the requisite training and certification. To ascertain the accuracy and consistency, the Shepherd Research Lab conducted an exhaustive expert review of the participants’ scans.

The application of the DXA scan facilitates the quantification of bone and soft tissue throughout the entirety of the human body. By employing this advanced imaging modality, precise skeletal measurements about the lumbar spine can be obtained, thereby enabling the determination of the lumbar BMD value. The software of scan analysis precisely delineated the boundaries of VAT and SAT, accurately quantifying their respective areas, mass, and volume within the approximate region of the L4 and L5 vertebrae.

The ascertained masses of VAT and SAT were subjected to conversion, resulting in the derivation of two distinct indices: the visceral mass index (VMI) and the subcutaneous mass index (SMI). These indices served as independent variables in this study. VMI was calculated by dividing the mass of visceral fat (kg) by the square of an individual’s height (m^2^), while SMI was determined by dividing the mass of subcutaneous fat (kg) by the square of the individual’s height (m^2^).

### Covariates

In collecting information about participants’ demographics and lifestyles, standardized questionnaires were used. Age was recorded during the screening process. As for race, participants were categorized as Mexican Americans, Other Hispanics, Non-Hispanic Whites, Non-Hispanic Blacks, and individuals of Other Races, including those who identify as Multi-Racial. To capture the educational background of the participants, their academic achievements were classified into three tiers: individuals with less than a high school diploma, high school graduates, and those who surpassed the high school level. Height and weight were measured according to the standard scheme by proficient researchers. Body mass index (BMI) was derived by dividing weight (kg) by the square of standing height (m^2^). Smoking status was classified into three distinct categories: never, ever, and current smokers. The physical activity questionnaire was administered to assess the participants’ specific types of activities, and their intensity, and subsequently determine the activity-specific metabolic equivalent task (MET) value. MET, serving as a metabolic equivalent, signified the ratio between the metabolic rate during a specific activity and metabolic rate at rest. In accordance with NHANES recommendations, the weekly MET values were calculated as follows: (8.0 MET score × minutes of vigorous work-related activity) + (4.0 MET score × minutes of moderate work-related activity) + (4.0 MET score × minutes of walking or bicycling trips) + (8.0 MET score × minutes of vigorous recreational physical activity) + (4.0 MET score × minutes of moderate recreational or leisure physical activity).Consequently, participants were divided into low and high physical activity categories based on their Met value (≤ 500Met/week or > 500Met/week)^18^. Hypertension was defined as the presence of mean systolic or diastolic blood pressure exceeding 140/90 mm Hg in three consecutive measurements or the use of prescribed antihypertensive medication. Diabetes was defined by participants with a glycosylated hemoglobin (HbA1c) level ≥ 6.5% or the utilization of diabetes medication. Serum samples of total calcium, phosphorus, and vitamin D were carefully collected, appropriately stored, and subsequently transported to the University of Minnesota Advanced Research and Diagnostic Laboratory for analysis. Detailed instructions for specimen collection and handling were followed in accordance with the NHANES Lab Procedure Manual.

### Statistical analysis

Following a designed stratified, multi-stage probabilistic sampling methodology, sample weights were incorporated into all analyses conducted. To provide a comprehensive description of the subjects’ demographic information, we represented continuous variables in the subjects demographic information as mean ± standard deviation (SD), and used a weighted linear regression model to calculate *P* values. Categorical variables were expressed as percentages, and *P* values were calculated using a weighted chi-square test.

A weighted multiple linear regression model was established to conduct an analysis of the relationship between VMI, SMI and lumbar BMD. To estimate the direction and magnitude of the effect, we provided standard coefficients (β) and 95% confidence intervals (CI). In the analysis, three different models were employed, each accounting for specific adjustments to address potential confounding factors. Model 1 did not include any confounding factor adjustments. Model 2 incorporated adjustments for age and gender, recognizing their potential impact on the relationship under investigation. Lastly, Model 3 aimed to account for all relevant confounding factors, providing a more comprehensive understanding of the relationship between VMI, SMI, and lumbar BMD. To further explore potential nonlinear correlation between the variables, smooth curve fitting techniques were employed. Additionally, a two-stage linear regression model was utilized to identify potential inflection points in the relationship between these variables.

All analyses were performed using R software, specifically version 3.6.3, as well as EmpowerStats software available at https://www.empowerstats.com. *P* values less than 0.05 are statistically significant.

## Results

### Participant characteristics

The characteristics of participants were analyzed according to lumbar BMD quartiles, which were classified as follows: Q1 (0.56–0.932 g/cm^2^), Q2 (0.932–1.027 g/cm^2^), Q3 (1.027–1.131 g/cm^2^), and Q4 (1.131–2.477 g/cm^2^) (Table 1). The results indicated significant differences among the different lumbar BMD quartiles concerning various factors, including age, gender, race, education levels, BMI, smoking status, physical activity, serum total calcium, phosphorus and vitamin D levels, hypertension, diabetes, and VAT mass. Specifically, Participants with lumbar BMD values in the lowest quartile tended to be older Caucasian with lower levels of education and physical activity, as well as serum vitamin D. Furthermore, this group exhibited higher rates of smoking, hypertension, and higher mass of SAT and VAT.

**Table 1:** The characteristics of participants included in this study.

Lumbar BMD (g/cm^2^)	Q1 (0.56–0.932)	Q2 (0.932–1.027)	Q3 (1.027–1.131)	Q4 (1.131–2.477)	*P* value
Age (years)	41.37 ± 11.73	38.46 ± 11.64	37.99 ± 11.43	38.63 ± 11.50	< 0.0001
Gender (%)					< 0.0001
Male	57.77	52.11	49.88	51.08	
Female	42.23	47.89	50.12	48.92	
Race (%)					< 0.0001
Mexican American	14.54	12.01	10.07	6.03	
Other hispanic	9.07	8.05	6.43	6.18	
Non-hispanic White	59.42	59.88	62.85	59.58	
Non-hispanic Black	6.01	9.14	11.99	19.33	
Other race	10.96	10.92	8.65	8.87	
Education level (%)					< 0.0001
Lower than high school	18.08	13.67	11.28	10.47	
High school	22.9	22.76	21.2	20.69	
More than high school	59.02	63.57	67.53	68.85	
Body mass index (kg/m^2^)	28.90 ± 6.36	28.81 ± 6.49	28.63 ± 6.84	29.45 ± 7.21	< 0.0001
High physical activity (%)					< 0.0001
No	14.48	13.6	11.84	12.56	
Yes	85.52	86.4	88.16	87.34	
Smoke (%)					0.0019
Never	55.93	59.67	61.21	61.11	
Ever	22.03	19	18.49	18.05	
Current	22.04	21.33	20.3	20.83	
Diabetes (%)					0.0022
No	93.27	94.79	94.02	91.93	
Yes	6.73	5.21	5.98	8.07	
Hypertension (%)					0.001
No	83.14	86.74	87.35	84.47	
Yes	16.87	13.26	12.65	15.52	
Total calcium (mmol/L)	2.34 ± 0.09	2.34 ± 0.08	2.34 ± 0.08	2.34 ± 0.08	0.9584
Phosphorus (mmol/L)	1.20 ± 0.18	1.19 ± 0.18	1.20 ± 0.18	1.20 ± 0.18	0.3654
Vitamin D (nmol/L)	65.01 ± 25.73	65.74 ± 25.51	66.96 ± 26.45	66.76 ± 26.46	0.0232
SATM (kg)	1.65 ± 0.77	1.64 ± 0.81	1.61 ± 0.85	1.62 ± 0.84	0.2021
VATM (kg)	0.57 ± 0.28	0.50 ± 0.28	0.47 ± 0.26	0.47 ± 0.30	< 0.0001

SATM, Subcutaneous adipose tissue mass, VATM, Visceral adipose tissue mass.

### Association between VMI and lumbar BMD

**Figure 2:**
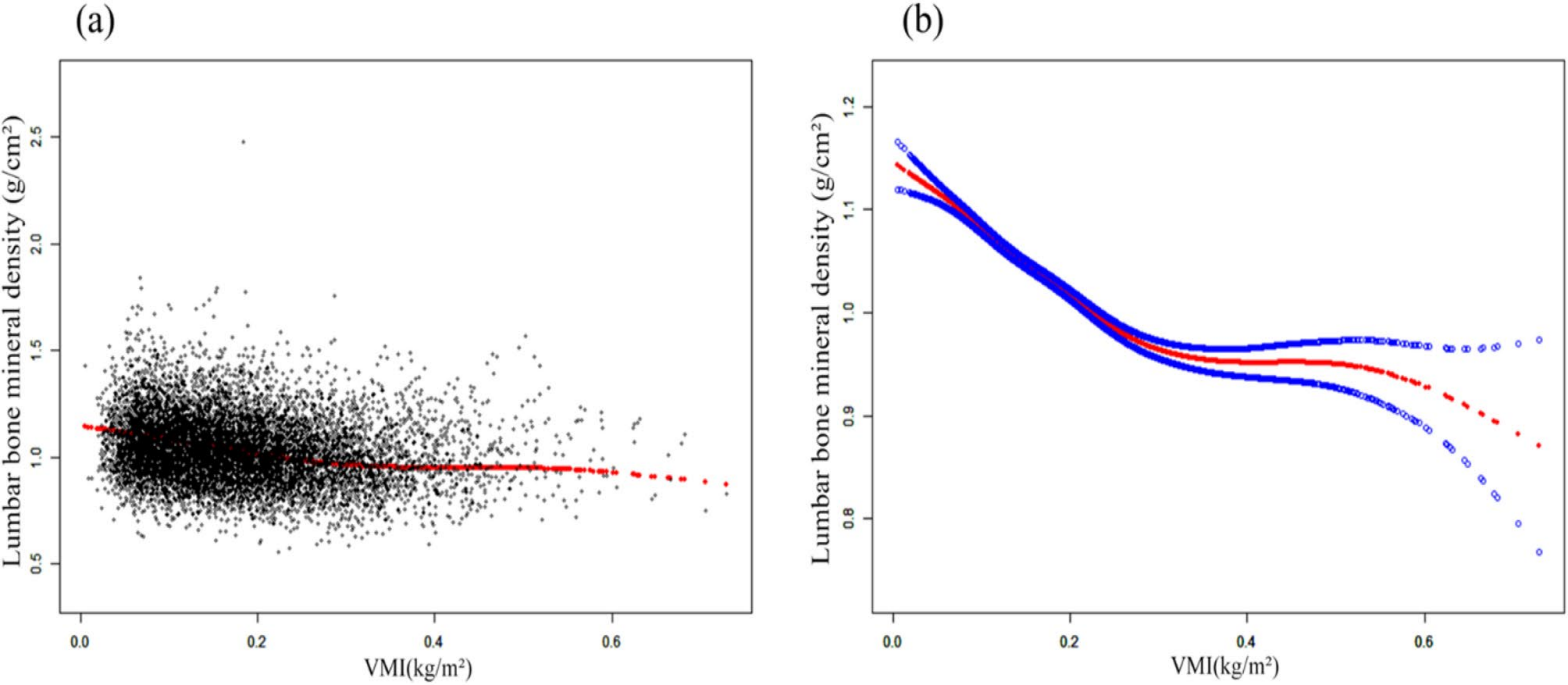
The association between VMI and lumbar bone mineral density. (**a**) Each black point represents a sample. (**b**) Solid rad line represents the smooth curve fit between variables. Blue bands represent the 95% of confidence interval from the fit. Adjusted for age, gender, race, education level, body mass index, smoke, physical activity, hypertension , diabetes , serum total calcium, serum phosphorus, serum vitamin D.

The association between VMI and lumbar BMD was investigated through three weighted multiple linear regression models (Table 2). In the unadjusted model(Model 1), a negative correlation was observed [β = − 0.231, 95% CI (− 0.260, − 0.203)], which persisted in the adjusted model 2 [β = − 0.222, 95% CI (− 0.254, − 0.191)] and model 3 [β = − 0.447, 95% CI (− 0.493, − 0.400)]. Compared to participants with the lowest VMI level in quartile 1 (Q1), participants in the other quartiles exhibited lower BMD values. Furthermore, as VMI increased, the more negative impact on BMD was observed. Using smooth curve fitting analysis, a “U” shaped association between VMI and BMD was identified (Fig. 2). Subsequently, a two-stage linear regression model calculated the inflection point as 0.304 kg/m^2^ (Table 3). In subgroup analysis stratified by BMI and gender, the “U” shaped relationship between VMI and BMD was observed specifically among men and individuals classified as obese (BMI ≥ 30 kg/m^2^). (Figs. 4 and 5).

**Table 2:** Association of SMI and VMI with lumbar bone mineral density.

	Model 1	Model 2	Model 3
	β (95% CI) *P* value	β (95% CI) *P* value	β (95% CI) *P* value
SMI (kg/m^2^)	− 0.025 (− 0.034, − 0.016) < 0.00001	− 0.038 (− 0.048, − 0.027) < 0.00001	− 0.310 (− 0.334, − 0.285) < 0.00001
Q1 (0.038–0.356)	Reference	Reference	Reference
Q2 (0.356–0.535)	− 0.020 (− 0.027, − 0.012) < 0.00001	− 0.019 (− 0.027, − 0.011) < 0.00001	− 0.034 (− 0.043, − 0.026) < 0.00001
Q3 (0.535–0.778)	− 0.026 (− 0.034, − 0.018) < 0.00001	− 0.030 (− 0.039, − 0.022) < 0.00001	− 0.065 (− 0.075, − 0.055) < 0.00001
Q4 (0.778–2.171)	− 0.023 (− 0.031, − 0.015) < 0.00001	− 0.033 (− 0.042, − 0.023) < 0.00001	− 0.118 (− 0.133, − 0.103) < 0.00001
VMI (kg/m^2^)	− 0.231 (− 0.260, − 0.203) < 0.00001	− 0.222 (− 0.254, − 0.191) < 0.00001	− 0.447 (− 0.493, − 0.400) < 0.00001
Q1 (0.005–0.099)	Reference	Reference	Reference
Q2 (0.099–0.159)	− 0.018 (− 0.025, − 0.010) 0.00001	− 0.017 (− 0.025, − 0.009) 0.00005	− 0.035 (− 0.043, − 0.026) < 0.00001
Q3 (0.159–0.233)	− 0.040 (− 0.048, − 0.033) < 0.00001	− 0.039 (− 0.047, − 0.031) < 0.00001	− 0.067 (− 0.077, − 0.057) < 0.00001
Q4 (0.233–0.73)	− 0.069 (− 0.077, − 0.062) < 0.00001	− 0.069 (− 0.077, − 0.060) < 0.00001	− 0.119 (− 0.131, − 0.108) < 0.00001

SMI, Subcutaneous mass index, VMI, Visceral mass index.

Model 1: No covariates were adjusted.

Model 2: Adjusted for age and gender.

Model 3: Adjusted for age, gender, race, education level, body mass index ,smoke, physical activity, hypertension , diabetes , serum total calcium, serum phosphorus, serum vitamin D.

**Table 3:** Threshold effect analysis of SMI and VMI on lumbar bone mineral density by using two-piecewise linear regression.

	Adjusted ß (95% CI), *P*-value
VMI	
Total	
Fitting by standard linear model	− 0.447 (− 0.493, − 0.400) < 0.0001
Fitting by standard linear model	
Inflection point	0.304
VMI < 0.304(kg/m^2^)	− 0.624 (− 0.680, − 0.568) < 0.0001
VMI > 0.304(kg/m^2^)	0.016 (− 0.080, 0.111) 0.7476
Log likelihood ratio	< 0.001
Male	
Fitting by standard linear model	− 0.554 (− 0.628, − 0.479) < 0.0001
Fitting by standard linear model	
Inflection point	0.301
VMI < 0.301(kg/m^2^)	− 0.766 (− 0.854, − 0.678) < 0.0001
VMI > 0.301(kg/m^2^)	0.115 (− 0.052, 0.283) 0.1763
Log likelihood ratio	< 0.001
BMI ≥ 30 kg/m^2^	
Fitting by standard linear model	− 0.207 (− 0.263, − 0.150) < 0.0001
Fitting by standard linear model	
Inflection point	0.297
VMI < 0.297(kg/m^2^)	− 0.477 (− 0.568, − 0.387) < 0.0001
VMI > 0.297(kg/m^2^)	0.119 (0.017, 0.221) 0.0222
Log likelihood ratio	< 0.001
SMI	
Total	
Fitting by standard linear model	− 0.310 (− 0.334, − 0.285) < 0.0001
Fitting by standard linear model	
Inflection point	0.203
SMI < 0.203 (kg/m^2^)	− 0.413 (− 0.543, − 0.284) < 0.0001
SMI > 0.203(kg/m^2^)	− 0.307 (− 0.332, − 0.282) < 0.0001
Log likelihood ratio	0.109

Age, gender, race, education level, body mass index (BMI), smoke, physical activity, hypertension, diabetes, serum total calcium, serum phosphorus, serum vitamin D were adjusted. In the analysis for gender or BMI, the model is not adjusted for gender or BMI respectively.

### Association between SMI and lumbar BMD

**Figure 3:**
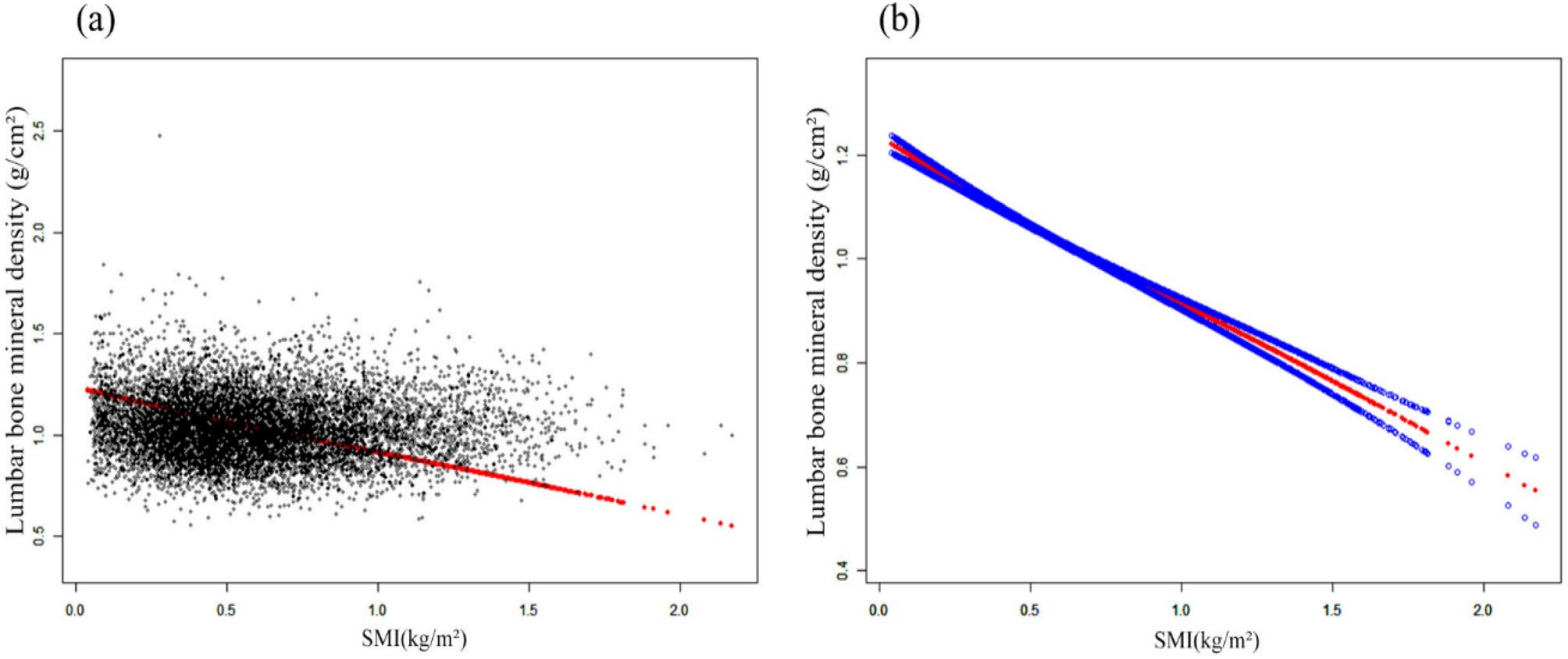
The association between SMI and lumbar bone mineral density. (**a**) Each black point represents a sample. (**b**) Solid rad line represents the smooth curve fit between variables. Blue bands represent the 95% of confidence interval from the fit. Adjusted for age, gender, race, education level, BMI, smoke, physical activity, hypertension, diabetes, serum total calcium, serum phosphorus, serum vitamin D.

**Figure 4:**
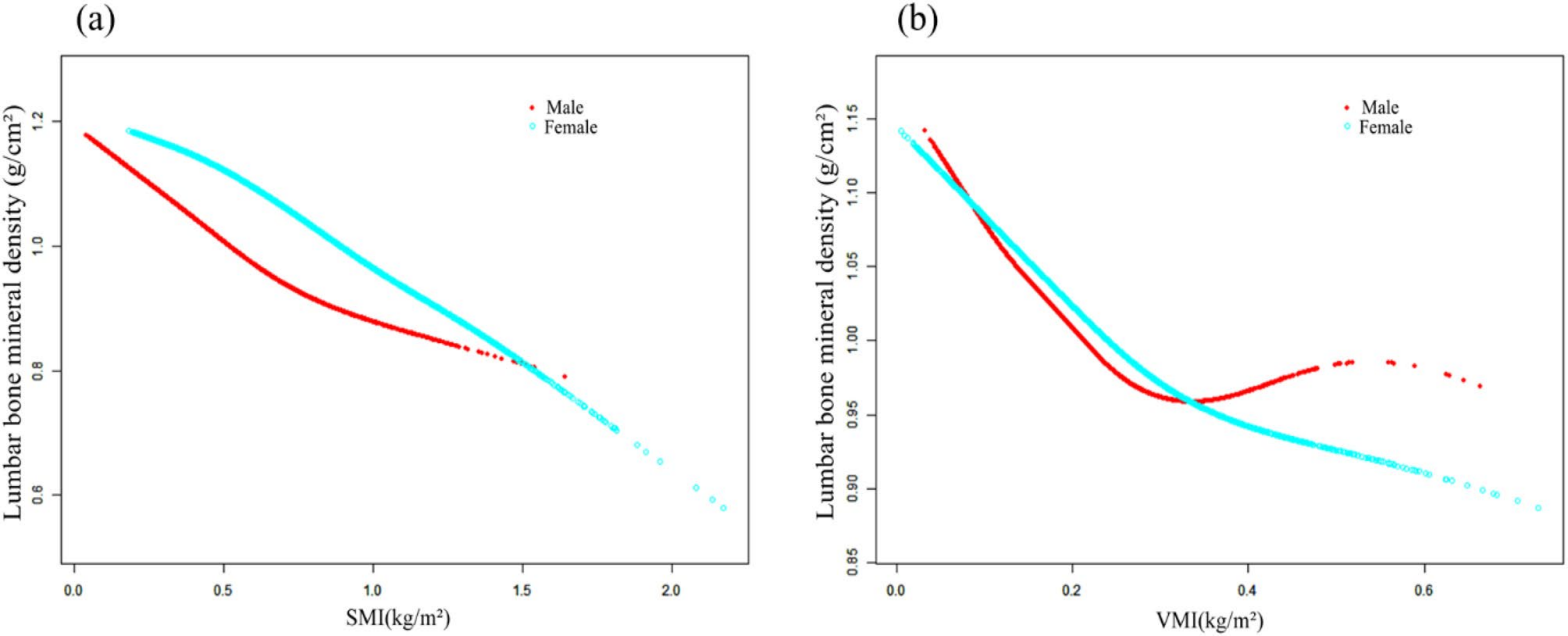
The associations between VMI (**a**), SMI (**b**) and lumbar bone mineral density stratified by gender. Adjusted for age, race, education level, body mass index, smoke, physical activity, hypertension, diabetes, serum total calcium, serum phosphorus, serum vitamin D.

**Figure 5:**
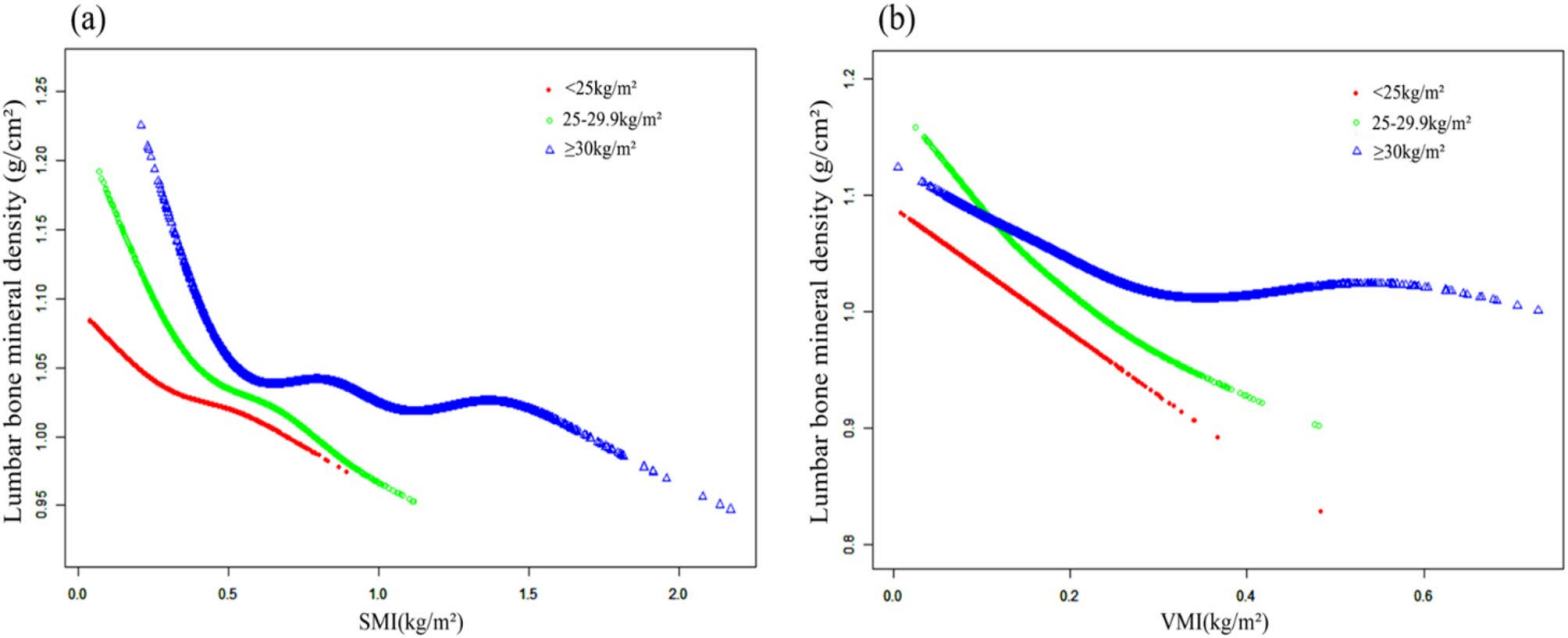
The associations between VMI (**a**), SMI (**b**) and lumbar bone mineral density stratified by body mass index (BMI). Adjusted for age, gender, race, education level, smoke, physical activity, hypertension, diabetes, serum total calcium, serum phosphorus, serum vitamin D.

The unadjusted model showed a negative correlation [β = − 0.025, 95% CI (− 0.034, − 0.016)], persistently endured within model 2 [β = − 0.038, 95% CI (− 0.048, − 0.027)] and model 3 [β = − 0.310, 95% CI (− 0.334, − 0.285)] (Table 2). Participants occupying the higher quartiles of SMI demonstrated a noticeable decrement in BMD when compared to those in the lowest quartile (Q1). Notably, the deleterious influence of SMI on BMD exhibited an escalating magnitude with ascending SMI levels. Employing the methodology of smooth curve fitting, we discovered the linear negative correlation between SMI and BMD (Fig. 3). Upon conducting a subgroup analysis predicated on gender and BMI stratification, we also unveiled the negative correlation between SMI and BMD (Figs. 4 and 5).

## Discussion

The primary objective of this study was to explore the relationship between the allocation of visceral or subcutaneous adipose tissue and BMD among individuals aged 20 to 59 years. The findings indicated there existed a negative connection between SMI and lumbar BMD. Furthermore, a U-shaped correlation emerged between VMI and lumbar BMD, with the inflection point at 0.304 kg/m^2^. However, this non-linear connection was observed exclusively among male participants or individuals with obesity.

VAT assumes a pivotal role in preserving human well-being by releasing fatty acids and hormones that wield significant influence over metabolism^19^. However, an excess accumulation of visceral fat has been linked to an elevated risk of chronic ailments^20,21^. Despite numerous studies, the association between VAT and bone health remains controversial. The majority of investigations have discovered a connection between increased visceral fat and reduced BMD^22–24^. In a community-based study of Australians aged 45–70 years, visceral fat was found to potentially exert detrimental effects on bone health, particularly among males^25^. Nevertheless, the Framingham Osteoporosis study^26^ found the positive connection between higher VAT levels and BMD, although this association lost significance after adjusting for BMI or weight. Similarly, a Chinese study failed to identify a discernible correlation between VAT and BMD^16^. However, an independent, positive connection between VAT and BMD was found by the Amirkola Health and Aging Study^27^. Our study, involving adults at a younger age compared to prior investigations, revealed a non-linear (U-shaped) relationship between VMI and lumbar BMD. This finding aligns with the reported connection between VAT and overall fracture risk as documented in observational and Mendelian randomization studies^28^. Furthermore, a meta-analysis^29^ demonstrated that a low BMI was associated with an elevated risk of fractures, but the connection shifted to a non-linear (U-shaped) pattern when comparing high BMI to normal BMI. A study by Andrea Palermo et al.^30^ exploring the connection between obesity and bone fragility indicated that an increasing BMI’s protective effect on fractures weakens within a specific range. However, with severe obesity, this impact tended to diminish. In light of these findings, we hypothesized that the accumulation of visceral fat might exert a major impact on the association between BMI and fracture risk.

The intricate and multifaceted interplay between visceral fat and the skeletal system involves several factors. Prominent proinflammatory cytokines emanating from visceral fat, such as IL-1,6 and TNF-α, engender a systemic inflammatory response, disrupt the metabolic equilibrium, and inflict deleterious consequences on bone health^31,32^. Adipocytokines, including leptin and adiponectin, wield a direct impact on skeletal metabolism. The correlation between Leptin and BMD has been observed to exhibit both negative and positive associations^33,34^. Leptin, in its capacity, can promote the differentiation of osteoblasts^35^. However, through its modulation of the sympathetic nervous system and cocaine-amphetamine regulated transcript, it may concurrently hinder bone growth^36^. Similarly, adiponectin appears to influence visceral fat and osseous metabolism in a manner that oscillates between favorable and unfavorable outcomes^37,38^. Via the MAPK signaling system, adiponectin can promote the proliferation and development of human osteoblasts^39^. Yet, by promoting RANKL and inhibiting the production of osteoblast osteoprotegerin, it can indirectly enhance the formation of osteoclasts, resulting in a decline in BMD^40^. Moreover, the metabolism of visceral fat and insulin resistance have a strong relationship^41,42^. Studies have shown that insulin-like growth factor-1 (IGF-1) has a positive correlation with BMD and a negative correlation with VAT. By influencing bone development, insulin resistance may exert control over the negative impacts of VAT on bone health^43^. However, given the dichotomous nature of these aforementioned factors, which may bestow both beneficial and detrimental effects on bone health, the consequences of these factors are still debatable. Therefore, further investigations are imperative to elucidate comprehensively the intricate nexus connecting between visceral fat and bone health.

The relationship between subcutaneous fat and bone health has been a subject of debate. The results stemming from various studies have engendered conflicting perspectives. Several studies^44,45^, such as the Older Afro-Caribbean Men study and a study involving older women, have found that higher amounts of SAT are associated with increased BMD, suggesting a protective effect on bone health. Subcutaneous fat may help with bone strength in healthy young women according to research by Vicente Gilsanz et al.^22^ However, research encompassing Chinese women failed to discern any discernible link between SAT and BMD^46^. Limited sample sizes and subjects who are mainly children, teenagers, or geriatric populations constitute two notable limitations afflicting these investigations. Our study revealed a negative relationship between subcutaneous fat and BMD, which is consistent with the findings reported by Katzmarzyk et al.^47^ and Wang et al.^48^.

Research findings have lent support to the proposition that the association between adipose tissue and BMD manifests variations contingent on the specific type of fat^49^. The cytokines, hormones, and inflammatory substances that can be excreted by adipose tissue exert influence over a diverse range of cellular processes. However, depot-specific variations in gene translation can engender an array of health ramifications. For instance, visceral adipocytes exhibit heightened resistance to insulin in comparison to subcutaneous adipocytes and possess a more active metabolism profile and greater lipolysis toxicity^50^. These two distinct adipose tissue exhibit differential secretion patterns of adipocytokines. SAT shows a greater expression of leptin and adiponectin in comparison to VAT^51^. Furthermore, VAT and SAT instigate disparate inflammatory states. Significantly more pivotal pro-inflammatory genes are expressed in SAT compared to VAT. In cases of extreme obesity, SAT assumes greater significance than VAT in promoting an environment conducive to inflammation^52^. Therefore, when investigating the impact of adipose tissue on human health, it becomes imperative to consider the specific adipose tissue type.

Individuals exhibiting a particular range of visceral adiposity, coupled with elevated levels of subcutaneous adipose tissue, face an augmented susceptibility to osteoporosis. The formulation of targeted screening and intervention strategies tailored to these high-risk cohorts holds the potential for early detection and enhanced management of bone health. Therefore, when evaluating body composition and its impact on bone health, due consideration must be given to both visceral and subcutaneous fat reservoirs. Prospective investigations could delve into the construction and validation of refined body composition assessment methodologies, enabling accurate quantification of visceral and subcutaneous fat depots. These advances would facilitate the precise stratification of risks and the monitoring of bone health. Moreover, future investigations should be undertaken to elucidate the impact of the observed correlation between visceral and subcutaneous adipose tissue and BMD on fracture susceptibility, bone remodeling processes, and holistic skeletal well-being across an extended temporal span. By conducting longitudinal studies that meticulously evaluate alterations in the distribution of adipose tissue and its intricate interplay with skeletal outcomes, an array of invaluable insights can be gleaned regarding the trajectory of bone health and potential junctures for targeted interventions throughout an individual’s lifespan.

The utilization of a large sample size and the incorporation of the up-to-date DXA data bestow a distinct advantage to this study. Meanwhile, our investigation offers important insights into the relationship between visceral and subcutaneous fat and BMD in adult populations. The inclusion of a representative sample encompassing multi-ethnic groups permits the generalizability of the findings to the broader population. However, several limitations must be taken into account while interpreting the findings. First off, Our study utilized a cross-sectional research design, which inevitably limits the ability to prove causality. Future investigations should adopt a longitudinal cohort design, encompassing a substantial sample size and an extended observation period, to validate these findings. Secondly, Despite our best efforts, it is important to acknowledge that the association between visceral and subcutaneous fat and BMD in American adults may still be confounded by other factors that we did not account for. Moreover, the self-reported data on medical history, smoking, and physical activity may be susceptible to memory bias. In addition, the presence of missing data introduces the possibility of biases and may affect the generalizability of the findings. Finally, the specific measurement of adipokines or cytokines was not conducted in our study, which could have provided valuable insights into the underlying mechanisms through which visceral fat and subcutaneous fat influence BMD.

## Conclusions

Our findings suggested that there existed distinct relationships between visceral fat, subcutaneous fat and bone health. Specifically, we found a U-shaped relationship between visceral fat and BMD, while subcutaneous fat exhibited a negative relationship with BMD. However, further investigation was warranted to gain a deeper understanding of the underlying mechanisms driving these relationships.

## Data Availability

The datasets generated during the current study are available in the NHANES repository (http://www.cdc.gov/nchs/nhanes.htm). The datasets generated and analyzed during the current study are available in the ZENODO repository, https://doi.org/10.5281/zenodo.7796587.
